# Comparative Genomics Identifies Novel Genetic Changes Associated with Oxacillin, Vancomycin and Daptomycin Susceptibility in ST100 Methicillin-Resistant *Staphylococcus aureus*

**DOI:** 10.3390/antibiotics12020372

**Published:** 2023-02-11

**Authors:** Sabrina Di Gregorio, María Sol Haim, Ángela María Rosa Famiglietti, José Di Conza, Marta Mollerach

**Affiliations:** 1 Instituto de Investigaciones en Bacteriología y Virología Molecular (IBaViM), Facultad de Farmacia y Bioquímica, Universidad de Buenos Aires, Ciudad Autónoma de Buenos Aires 1113, Argentina; 2 Consejo Nacional de Investigaciones Científicas y Técnicas (CONICET), Ciudad Autónoma de Buenos Aires 1113, Argentina; 3 Unidad Operativa Centro Nacional de Genómica y Bioinformática, ANLIS Dr. Carlos G. Malbrán, Ciudad Autónoma de Buenos Aires 1282, Argentina; 4 Laboratorio de Bacteriología Clínica, Hospital de Clínicas José de San Martín, Facultad de Farmacia y Bioquímica, Universidad de Buenos Aires, Ciudad Autónoma de Buenos Aires 1113, Argentina

**Keywords:** *S. aureus*, hVISA/VISA, MRSA, WGS, ST100, IS*256*

## Abstract

Infections due to vancomycin-intermediate *S. aureus* (VISA) and heterogeneous VISA (hVISA) represent a serious concern due to their association with vancomycin treatment failure. However, the underlying molecular mechanism responsible for the hVISA/VISA phenotype is complex and not yet fully understood. We have previously characterized two ST100-MRSA-hVISA clinical isolates recovered before and after 40 days of vancomycin treatment (D1 and D2, respectively) and two in vitro VISA derivatives (D23C9 and D2P11), selected independently from D2 in the presence of vancomycin. This follow-up study was aimed at further characterizing these isogenic strains and obtaining their whole genome sequences to unravel changes associated with antibiotic resistance. It is interesting to note that none of these isogenic strains carry SNPs in the regulatory operons *vraUTSR*, *walKR* and/or *graXRS*. Nonetheless, genetic changes including SNPs, INDELs and IS*256* genomic insertions/rearrangements were found both in in vivo and in vitro vancomycin-selected strains. Some were found in the downstream target genes of the aforementioned regulatory operons, which are involved in cell wall and phosphate metabolism, staphylococcal growth and biofilm formation. Some of the genetic changes reported herein have not been previously associated with vancomycin, daptomycin and/or oxacillin resistance in *S. aureus*.

## 1. Introduction

*Staphylococcus aureus* is a relevant pathogen with an extraordinary ability to evolve and acquire resistance to several antibiotics. Over the last decades, the large spread of antimicrobial-resistant (AMR) strains, including methicillin-resistant *S. aureus* (MRSA), vancomycin-intermediate *S. aureus* (VISA) and heterogeneous VISA (hVISA), have raised an alarm worldwide as declared by the World Health Organization in 2017 [1]. hVISA/VISA isolates are associated with persistent infections, vancomycin treatment failure and poor clinical outcomes [2].

Although the prevalence of hVISA and VISA is relatively low worldwide [3,4,5], a recent review and meta-analysis revealed that it has been increasing since 2010 (especially in Asia and America) [3]. This highlights the importance of understanding the resistance mechanism in order to define adequate control measures.

It has been twenty-five years since the first hVISA/VISA strains were reported [6,7]. However, the underlying molecular mechanism responsible for the hVISA/VISA phenotype is not yet fully understood. Moreover, the reduced susceptibility to vancomycin is often accompanied by concomitant changes in the susceptibility to oxacillin [8,9] and other last-resort antibiotics such as daptomycin [10], making it more difficult to establish a correct treatment for infections caused by these strains. Whole genome sequencing (WGS) of hVISA/VISA strains has been essential for detecting genetic changes associated with their phenotype. Despite the evidence showing modifications in peptidoglycan metabolism in hVISA/VISA, associated genetic changes seem to implicate a diverse set of mutations and chromosomal rearrangements. Non-synonymous single nucleotide polymorphisms (SNPs) in *vraSR*, *yvqF/vraT*, *walKR*, *graXRS* (involved in peptidoglycan metabolism and cell wall stress stimulon) or *rpoB* were amongst the first and most frequently reported genetic changes in hVISA/VISA [11,12,13]. Mutations in those genes have been experimentally tested to be responsible for promoting vancomycin resistance in VISA. In addition, some researchers reported IS*256* insertions disrupting different genes implied in cell wall synthesis (*tcaA*, *walKR*) that lead to the VISA phenotype [14,15,16,17] and daptomycin resistance [18].

IS*256* is an insertion sequence that has been detected in multiple copies in the genome of *Staphylococcus* spp. strains recovered from humans and animals [19,20]. IS*256* can be found flanking the ends of transposon Tn*4001* [21], and it has been prevalently described in MRSA clones belonging to CC8 (ST239, ST247, ST8) and CC5 (ST5, ST100, ST228) [16,18,22,23,24]. The transposition of IS*256* in *S. aureus* is a copy-and-paste mechanism [25] and can result in a variety of genetic modifications that affect the expression of genes involved in virulence and antimicrobial resistance [26,27,28,29]. Our results highlighted that vancomycin treatment increased IS*256* transposition and showed that different VISA phenotypes could be selected from the same parental ST100-hVISA strain [24,30].

In this follow-up study, our aim is to further characterize these isogenic strains and to obtain their whole genome sequences in order to unravel genetic changes associated with antibiotic resistance.

We herein describe novel mutations and genetic rearrangements developed after vancomycin pressure (in vivo treatment and in vitro selection), some of which (as far as we know) have not been previously reported in the literature as associated with vancomycin, daptomycin and/or oxacillin resistance in *S. aureus*.

## 2. Results

### 2.1. Antimicrobial Susceptibility

**Figure 1:**
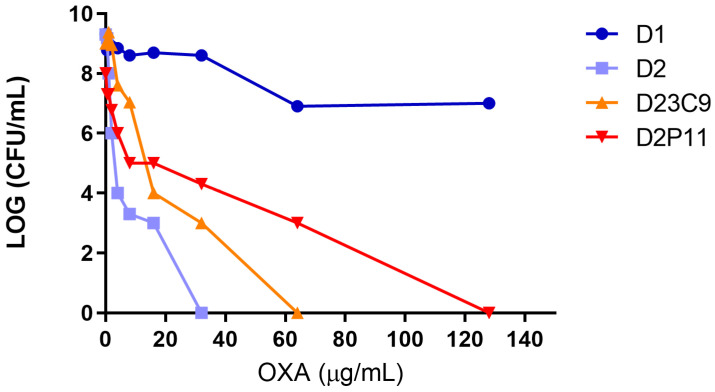
Oxacillin population analysis profile and area under the curve (PAP-AUC) of bacterial strains included in this study. Serial 10-fold dilutions of cultures were plated onto Mueller–Hinton agar with oxacillin (OXA). Each point represents the viable count (log10 CFU/mL) after 48 h against increasing concentrations of OXA.

ST100 strains D1, D2, D23C9 and D2P11 differed not only in their susceptibility to vancomycin, but also to oxacillin (Table 1). A deeper analysis revealed that D2, recovered after arthrotomy and surgical cleaning after 40 days of vancomycin treatment, significantly increases its oxacillin susceptibility when compared to D1, rendering a phenotype that resembles a heteroresistant behavior (Figure 1). In addition, VISA derivatives D23C9 and D2P11 presented a 4–16-fold and 1.31–2.63-fold increase in oxacillin and daptomycin MIC relative to parental strain D2, respectively (Table 1).

### 2.2. General Genomic Features

The assembled draft genomes of the four strains yielded the following results: total genome length of 2,785,355–2,795,159 pb; GC content of 32.79–32.81%; 31–40 contigs > 1 kb in length; and N50 of 148,220–337,832 bp. (Table 2). All strains harbor the *mecA* gene within the class B *mec* gene complex of a truncated *SCCmec* with no *ccr* genes (Table 3). An overview of mobile genetic elements and AMR determinants found is summarized in Supplementary Table S2.

### 2.3. Mutations Associated with hVISA/VISA

All 4 strains shared 20 non-synonymous SNPs in genes related to cell wall metabolism and/or the hVISA/VISA phenotype (Table 4) when compared to *S. aureus* N315 (listed in Supplementary Table S3). It is worth noting, none of the strains carried SNPs in *vraTSR*, *walKR* and/or *graXRS* operons.

Hence, comparative genomic analyses between D1 and D2, and between D2 and its in vitro-derived mutants were performed to unravel the genetic differences associated with their AMR profiles.

Seven SNPs and four INDELs distinguish D2 and its derived mutants (D23C9, D2P11) from D1 (recovered before vancomycin treatment) (Table 5). INDELs affected genes *stp1*, *braS*, *sagB* and *era*, related to cell stress and envelope metabolism [31,32,33,34,35] and possibly related to the different oxacillin and vancomycin phenotypes observed (Table 1, Figure 1).

**Figure 2:**
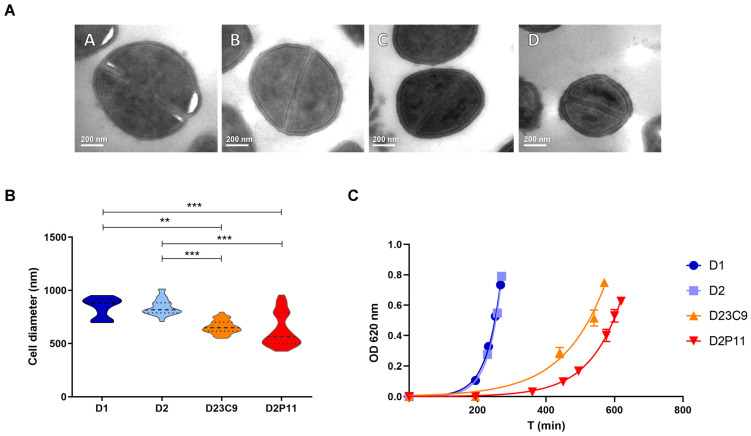
(**A**) Transmission electron microscopy (TEM) of representative cells; images obtained at 50,000×. A: D1; B: D2; C: D23C9; D: D2P11. (**B**) Cell diameter measured by TEM (30 cells per strain). The results are expressed in nanometers (nm) as violin plots showing median (dashed line) and interquartile range (dots) and were compared with Kruskal–Wallis’s test (*p* < 0.0001). The horizontal bars show significant differences between individual groups detected by Dunn’s multiple comparison test. ** *p* < 0.001; *** *p* < 0.0001. (**C**) Growth curves. Each point represents the mean and standard error from three independent assays.

In addition, in vitro-derived VISA strains D23C9 and D2P11, harbor mutations leading to premature stop codons in two genes linked to staphylococcal growth (*phoR* and *era*, respectively) (Table 5) [35,36]. Along with these genetic changes, both VISA strains showed a lower median cell diameter, longer latency growth phase and slower growth (slopes in the logarithmic exponential growth phase were significantly different, *p* < 0.0001), when compared to parental strain D2 (Figure 2).

### 2.4. IS256-Mediated Genomic Rearrangements

Changes in IS*256* transposition after vancomycin selective pressure for this set of strains [24] were also detected by WGS bioinformatic analysis performed in this study (Table 6 and Table 7).

Comparative genomic analysis of paired *S. aureus* strains showed evidence of genetic rearrangements after the IS*256* transposition-mediated antibiotic treatment. In total, 11 different IS*256* insertions sites (4/11 disrupting genes) were shared by the 4 strains (Table 7), while both in vitro-derived mutants (D23C9 and D2P11) showed modifications in the IS*256* copy number and position (Table 5). We did not find genetic changes in the *sigB* and *rsbU* genes, known global regulators of IS*256* transposition [26,37]. In addition, no change in the copy number and/or location of other staphylococcal IS elements was evident.

**Figure 3:**
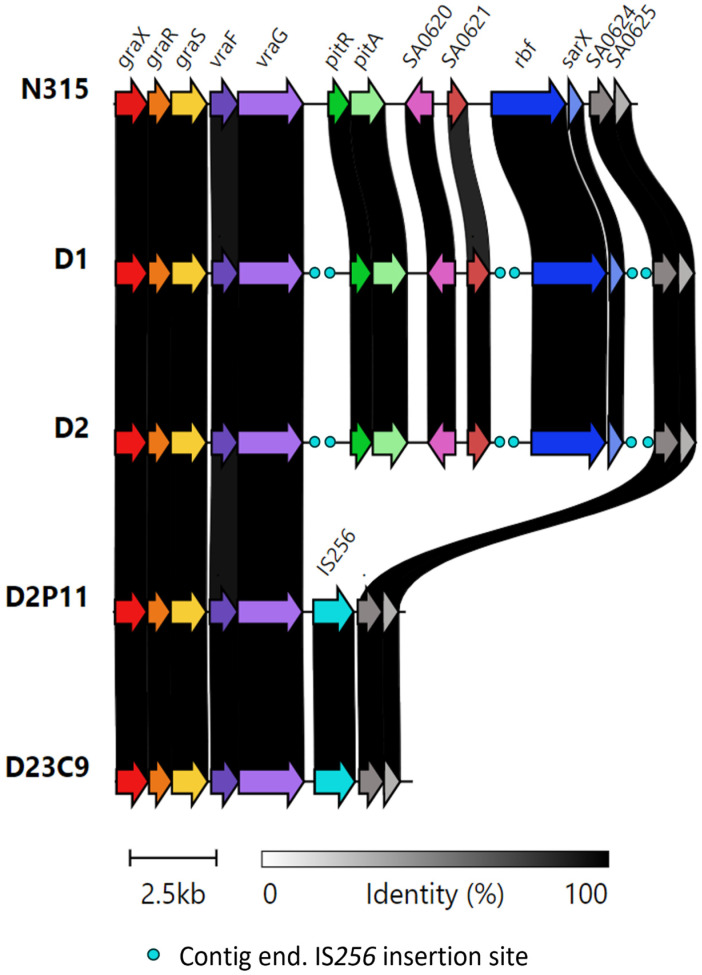
Clinker gene cluster comparison of the genomic region between *graX* and SA0625. Homologous CDSs are in the same color and linked through grey bars with the percentage amino acid identity, as indicated in the legend. IS*256* insertions are marked as aqua circles (in case of contig ends), or annotated CDS (confirmed by PCR).

It is worth noting that both VISA derivatives are characterized by the absence of a ≈ 8 kb region encompassing genes *pitR* (phosphate uptake regulator), *pitA* (low affinity inorganic phosphate transporter), SA0620 (secretory antigen ssA-like protein—CHAP domain peptidoglycan hydrolase), SA0621 (integral membrane protein interacts with ftsH-like protein), *rbf* (araC type transcriptional regulator) and *sarX* (transcriptional regulator). This was confirmed by the lack of raw reads mapping to the corresponding region in the assembled genome of parental strain D2, and by PCR–Sanger sequencing (Figure 3, Supplementary Table S1).

The presence of IS*256* insertion sites between *vraG-pitR*, SA0621-*rbf* and *sarX*-SA0624, in the D1 and D2 genomes, suggests that genetic rearrangements between neighboring IS*256* elements might be responsible for this region deletion in the in vitro-derived VISA strains.

We further evaluated if the ≈8 kb deletion and/or IS*256* insertion sites present in its genetic environment were shared by other strains. No other public genomes were found with the ≈8 kb deletion carried by D23C9 and D2P11 (BLAST searches against the NCBI Refseq complete *S. aureus* genomes database, last accessed 20 January 2023).

In line with previous results [24], IS*256* transposition was higher in D23C9. The genome of this strain contains four additional IS*256* insertion sites: one interrupting the *agrB* gene (already reported), and three newly reported (Table 7).

### 2.5. Biofilm Formation

**Figure 4:**
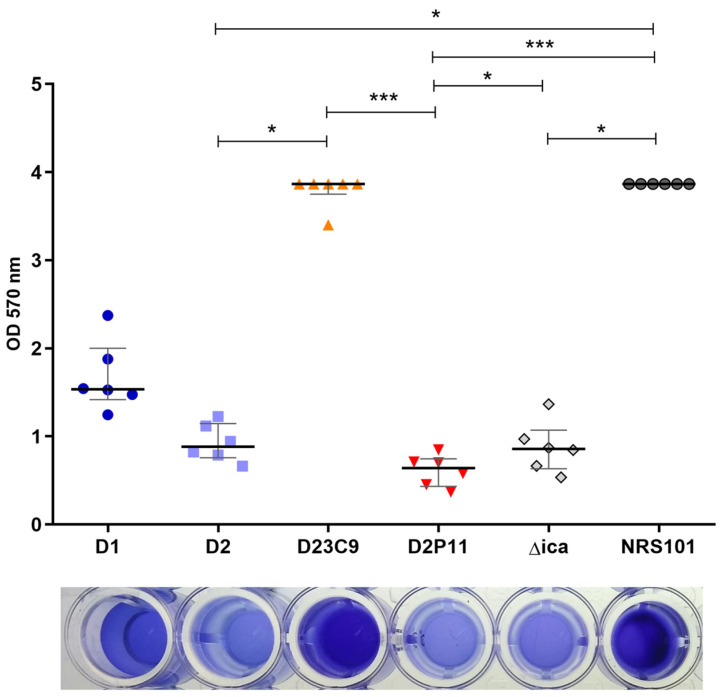
Biofilm production. Results are represented as final OD for each strain with median value (line) and interquartile range (whiskers) and are compared with Kruskal–Wallis’s test (*p* < 0.0001). The horizontal bars show significant differences between individual groups detected by Dunn’s multiple comparison test. * *p* < 0.05; *** *p* < 0.001. NRS101 (*S. epidermidis* NRS101), strong biofilm producer; Δ*ica* (*S. aureus* Newman Δ*ica*), *ica* independent-biofilm producer strain. The bottom picture shows a representative microtiter plate of the biofilm assay for each strain.

Knowing that *rbf*, *sarX* and *agrB* are involved in biofilm regulation [38,39], we studied the biofilm phenotype in these strains. D23C9 showed a significantly higher biofilm formation compared to parental strain D2 (Figure 4), and we speculated this is possibly due to both *agrB* disruption and extracellular DNA (product of D23C9 increased autolysis [24,30]. Moreover, D2P11 did not differ significantly from D2 in its ability to form biofilm despite the deletion of *rbf* and *sarX* genes but tends to display lower OD 570 nm values. However, small differences are not always detected on polystyrene microplates [40]; hence, microscopic changes affecting the three-dimensional biofilm structure should not be disregarded.

## 3. Discussion

Several single nucleotide polymorphisms (SNPs) have been described in hVISA/VISA strains since their first report, and new studies are still trying to understand their genetic basis [41,42]. Our findings reinforce the diversity of the genetic patterns observed [11,12,41], but also highlight the important role of INDELs and genomic rearrangements mediated by insertion sequences, particularly IS*256* in the emergence of these complex phenotypes.

**Figure 5:**
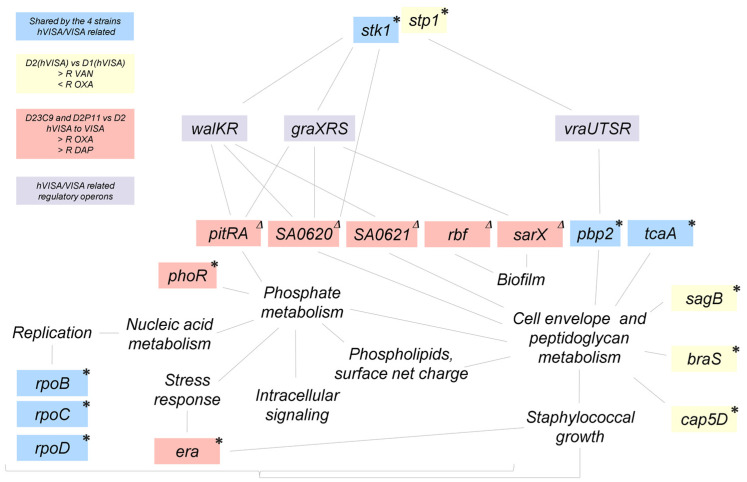
Genes harboring genetic changes in hVISA/VISA strains analyzed in this study and their relationships with cellular processes (lines). Blue boxes: Genes with mutations related to hVISA/VISA shared by D1, D2, D23C9 and D2P11. Yellow boxes: Genes with mutations differing between D2 and D1. Red boxes: Genes differing between the in vitro-selected mutants (D23C9 and D2P11) and their parental strain (D2). Δ ≈8 kb IS*256*-mediated deletion. * SNP/INDEL. Purple boxes: Key regulatory operons related to vancomycin resistance in hVISA/VISA (not mutated in these strains). R: Resistance. VAN: vancomycin. OXA: oxacillin. DAP: daptomycin.

The genetic changes found here could have resulted from the antibiotic selective pressure (in vivo and in vitro), but not all of them may necessarily have a direct correlation with the observed phenotypes. Evidence showed modifications in the peptidoglycan metabolism for hVISA/VISA strains and those analyzed in this study [11,30]. Mutations in genes *rpoB*, *rpoC*, *rpoD*, *pbp2*, *stk1* and *tcaA*, shared by the four strains, were also reported in clinical strains with vancomycin reduced susceptibility [11,12,43]. Nonetheless, we identified novel mutations in genes related to peptidoglycan metabolism (Table 4 and Table 5) in clinical hVISA strains (D1, D2). These novel mutations or other additional mutations related to different cellular processes (Supplementary Table S2) could have cumulative effects that contribute to hVISA/VISA, and/or oxacillin and daptomycin resistances, and should be experimentally investigated in future studies. However, mutations shared by all strains do not explain the phenotypic differences observed among them (Table 1, Figure 5) [30].

It is worth noting that two INDELs were exclusively found in hVISA strain D1. The *stp1* gene was associated with the reduced susceptibility to vancomycin, and the *braS* gene (alternatively named *nsaS* or *bceS*) is part of the *braRS* two-component system responding to cell envelope stress, which is referred to as *graS* ortholog (involved in hVISA/VISA phenotype) [34,44]. The in-frame deletion found in D1 (Gln63del) is located on the BraS cytoplasmic domain, next to the HisKA domain in charge of signal transduction and gene expression. We assume the hypothesis that these two genetic changes (Table 3), together with non-synonymous SNPs in genes related to cell wall metabolism (Table 2), could be involved in the vancomycin heteroresistance phenotype of D1. Nonetheless, these two INDELs are absent in the isogenic strain D2, possibly reflecting that different hVISA populations can be selected after vancomycin treatment. However, we recognize that as we sequenced just one colony from each clinical sample, we may have found a small proportion of all possible genetic changes, considering that hVISA strains are non-homogeneous populations. Nonetheless, our results show genetic changes that might contribute to antimicrobial resistance.

While the increase in oxacillin susceptibility was already reported in VISA strains [8,9], changes observed in this study are not due to *mecA*/*blaZ* mutations or deletions as described before. The *sagB* gene codes for the major glucosaminidase in charge of glycan chain processing in *S. aureus* (248aa) [33,45]. Its function, non-redundant despite the presence of other autolysins, is critical for cellular enlargement [33]. The frame-shift insertion shared by D2-D23C9-D2P11 generates a premature stop codon, and the predicted translated protein (148aa) lacks most of the glucosaminidase domain. SagB in vitro-selected mutants were described to display diminished resistance to oxacillin and increased resistance to vancomycin [45,46] as observed in D2 when compared to D1 (Table 1) [24].

No other mutations related to peptidoglycan metabolism/regulation were found between the D1 and D2 (*mecA*, *pbps*, *blaZ* operon and/or cell wall stimulon). Hence, it is most likely that genetic changes in *cap5D*, *stp1*, *braS* and/or *sagB* genes (related to peptidoglycan metabolism, Table 5) might play a role in the modification of cell wall thickness, *pbp2* expression, oxacillin and vancomycin susceptibility [24,30] between these two isogenic strains (Figure 5). As far as we know, this would be the first report on the acquisition of genetic changes in *braS* and *sagB* after the in vivo treatment with vancomycin, and its association with the hVISA phenotype and changes in oxacillin susceptibility in clinical strains.

Furthermore, one genetic change seems to be linked to the hVISA to VISA conversion. Both VISA strains selected in independent in vitro assays share not only the increase in oxacillin, vancomycin and daptomycin resistance, slower growth rate and reduced cell diameter (Table 1, Figure 1 and Figure 2), but also the IS*256*-mediated deletion of a ≈ 8 kb chromosomal region including regulatory genes related to metabolism (*pitRA*, SA0620, SA0621) and virulence (*rbf*, *sarX*) (Figure 3 and Figure 5). Genes related to the inorganic phosphate (Pi) metabolism (including *pitRA* and *phoR*) were previously associated with or reported to play a role in the development of vancomycin and daptomycin resistance [47,48,49,50], as changes in the intracellular Pi concentrations can affect the metabolism of DNA, phospholipids, cell envelope (including net positive surface charge), intracellular signalling and stress response [47,51]. Moreover, the expression of *pitRA*, SA0620, SA0621 and *sarX* is regulated by *walKR* and/or *graSR* operons involved in cell wall metabolism [43,52,53,54,55], and the development of vancomycin resistance in *S. aureus* [11]. The *rbf* gene was also frequently mutated in daptomycin-resistant *S. aureus* [56]. Together, all these findings highlight a potential relevance of the deleted genomic region for the development of oxacillin, vancomycin and daptomycin resistance in the ST100 genetic background.

Nonetheless, other SNPs or IS*256* rearrangements distinguishing D23C9 and D2P11 may also possibly contribute to their vancomycin resistance phenotype. In particular, mutations in genes associated with staphylococcal growth (*phoR* [36,47] and *era* [35], Table 5, Figure 5) may also impact on their growth rate and fitness cost (Figure 2), an already described feature of VISA isolates [11]. Interestingly, an INDEL in *era* was already described as a rare genetic change observed in a laboratory-derived VISA strain belonging to CC5 [50].

WGS is a powerful tool for the detection and surveillance of new AMR genetic determinants, but it has not been widely distributed in clinical laboratories yet, especially in low–middle income countries. Moreover, those who have access to this technology cannot depend solely on molecular assays to reliably detect all hVISA/VISA. Because of the multiplicity of genes involved, genomic approaches trying to establish only a few genetic markers as predictors of vancomycin heteroresistance will lead to an underestimation of the real prevalence, leaving behind new, unexplored hVISA phenotypes.

Nevertheless, studies supplementing whole genome sequences, the gold standard PAP-AUC method and MIC determinations are essential for detecting hVISA/VISA and other AMR phenotypes [50,57,58] until new approaches are developed.

Comparative omics analyses will help clarify the molecular mechanisms involved in the emergence of the hVISA/VISA phenotype in future research. Furthermore, the role of mutations (SNPs and INDELs) and the transposition of insertion sequences on the adaptation of *S. aureus* under antibiotic selective pressure should be explored. This work provides new evidence of the genetic rearrangements mediated by IS*256* transposition after antibiotic treatment, with the potential to impact the AMR and virulence of *S. aureus* strains.

## 4. Materials and Methods

### 4.1. Strains and Culture Conditions

*S. aureus* strains D1 and D2 were isolated from a patient with bone and joint infection, before and after 40 days of vancomycin treatment. The in vitro selection of D2-derived mutants (D23C9, D2P11) was performed in two independent assays by serial passage in BHI broth (Britania, Argentina) with increasing concentrations of vancomycin. D23C9 and D2P11 were selected at 9 and 11 µg/mL of vancomycin, respectively. The detailed medical record and the in vitro selection of D2-derived mutants (D23C9, D2P11) was previously described [24]. Strains were grown aerobically on Brain Heart Infusion (BHI) broth and BHI agar (Britania, Argentina) at 37 °C.

### 4.2. Antimicrobial Susceptibility Testing

Minimal inhibitory concentrations (MICs) of vancomycin (VAN) and oxacillin (OXA) were determined by the broth microdilution method according to CLSI guidelines [59]. Daptomycin susceptibility was evaluated by Etest^®^ and interpreted as per CLSI guidelines [59] and by the pre-diffusion method using Neo-Sensitabs^®^ tablets (Rosco Diagnostica, Taastrup, Denmark) [60,61]. The oxacillin and vancomycin population analysis profile and area under the curve (PAP-AUC) was determined as previously described [62]. ATCC 29213 (MSSA, VSSA), Mu3 (MRSA, hVISA) and Mu50 (MRSA, VISA) were used as control strains. The antimicrobial susceptibility profile is summarized in Table 1.

### 4.3. DNA Extraction and Whole Genome Sequencing

Genomic DNA was extracted from overnight BHI cultures using the Epicentre MasterPure Complete DNA and RNA Purification Kit according to the manufacturer’s instructions, with the addition of lysostaphin (0.03 µg/µL) in the lysis step with an incubation time of at least half an hour at 37 °C. Shotgun gDNA libraries were prepared and whole genome sequencing (WGS) was performed using the Illumina MiSeq platform (paired end, 250 bp).

### 4.4. Whole Genome Sequencing Analysis

Reads were quality assessed with FASTQC [63], and de novo assembled using SPAdes (v3.9.0) [64]. Contigs less than 500 bp and 70× coverage were discarded. Remaining contigs were annotated using Prokka (1.14.5) [65] and a genus-specific database from RefSeq [66], and they were manually inspected. Mapping and variant calling was carried out using Snippy v3.2 [67] with the following parameters: minimum quality of 30, minimum coverage of 15, minimum proportion of reads which must differ from the reference of 0.75. The genome of *S. aureus* N315 (CC5, Genebank Accession number BA000018.3) was used as a reference. Alternatively, the assembled genome of the first clinical isolate, D1 (ST100) was used as a reference sequence. All variants (SNPs, INDELs) were manually inspected and visualized with Artemis [68].

The *SCCmec* type was determined from assemblies using *SCCmec* Finder [69]. Detection of antimicrobial-resistance determinants and MGE was carried out using ARIBA v2.12.1 [70] and relevant databases. For antimicrobial-resistance determinants we used databases from Resfinder [71,72], CARD [73], ARGANNOT [74] and a curated database [75]. Plasmid types were defined based on their replicon genes (rep) using the Plasmidfinder database [76]. Phages types were defined based on their integrase gene, using the 12 described integrase groups [77]. Thirteen known staphylococcal pathogenicity islands (SaPIs) were queried based on their integrase (*int*) gene [78].

ISseeker [79] was used with default parameters to explore the genome in order to detect differences in insertion sequence (IS) content between parental and mutant strains, and also to annotate the flanking edges of IS elements in draft genomes. ISseeker identifies the termini of IS (>97% of identity) at contig edges and annotate flanking regions based on alignment of IS flanks with a reference genome. IS*256*-mediated insertions/deletions were confirmed by PCR with primers designed for that purpose (Supplementary Table S1). The 8 kb deletions amplified by PCR in VISA strains were sequenced by the Sanger method and analyzed with SnapGene v6.1.2^®^. Genomic comparisons were performed using Clinker [80]. The sequence of the 8 kb deletion was searched against the NCBI Refseq *S. aureus* complete genomes database using BLAST. All genomes, MGE and genome comparisons with reference sequences of interest were additionally visualized in Artemis and/or ACT [68,81].

### 4.5. Growth Curves

Growth curves were plotted to determine whether the genetic changes were associated with a fitness cost. These assays were performed by triplicate. Fresh culture of each strain (dilution 1/1000) was grown in BHI broth (Britania, Argentina) and incubated at 37 °C and 180 rpm, and OD 620 nm was measured. A growth curve was constructed plotting the OD 620 nm over time.

### 4.6. Transmission Electron Microscopy (TEM)

TEM of exponential phase *S. aureus* cultures was performed as already described [30]. Cell diameter was measured (30 cells for each strain) at the equatorial plane of each cell using a 50,000× magnification and images analyzed with ImageJ 1.46r [82]. The results for each strain were expressed as median and interquartile range.

### 4.7. Biofilm Production Assay

Biofilm development was assessed by measuring the accumulation of biomass on the surface of sterile 96-well flat-bottom polystyrene plates (Extragene) following Stepanovic et al. recommendations [83]. Briefly, 200 μL of a 1/100 dilution of a bacterial suspension adjusted to an OD 620 nm = 0.2 (≈108 CFU/mL) in TSB supplemented with sterile 1% glucose was added to wells (6 replicates per strain). Following 24 h incubation at 37 °C, the plate was washed twice with 0.9% NaCl and air-dried for 2 h. The remaining attached bacteria were fixed with 200 μL of methanol 99% (*v*/*v*) per well and after 15 min the plates were emptied and air-dried. Afterward the plates were stained for 20 min with 200 μL per well of 0.5% crystal violet. Finally, wells were washed with water, air-dried, the dye was solubilized with 33% acetic acid solution and the OD 570 nm for each well was measured. *S. aureus* Newman Δ*ica* (non-*ica*-dependent biofilm producer) and *S. epidermidis* NRS101 (prototype biofilm producer) were included in the assay as control strains. Biofilm production was calculated as Final OD 570 nm of a tested strain = average OD 570 nm value of the strain—ODc. ODc = average OD 570 nm of negative control Δ*ica* + 3SD of negative control.

### 4.8. Statistical Analysis

Growth rates were compared by slope analysis using linear regression. Biofilm production (Final OD 570 nm) and cell diameter values were compared using the Kruskal–Wallis’s test, and differences between individual groups were detected by Dunn’s multiple comparison test. Analyses were performed using GraphPad prism 5.0 software with a significance level set at *p* < 0.05 in all cases.

## Figures and Tables

**Table 1:** Antimicrobial susceptibility profile of the bacterial strains used in this study. MICs (µg/mL), inhibition zone diameter obtained after daptomycin (DAP) pre-diffusion method (mm), and vancomycin (VAN) population analysis profile and area under the curve (PAP-AUC) ratio. The AUC was measured for each sample and the ratio of test isolate AUC/mean Mu3 AUC was calculated. Mu3 was used as hVISA control strain. The criteria to define hVISA and VSSA were a PAP-AUC ratio ≥ 0.90 and a PAP/AUC ratio < 0.90, respectively.

		D1	D2	D23C9	D2P11	Reference
MIC (µg/mL)	VAN	0.5	1	4	8	[24]
	OXA	128	2	8	32	[24]
	DAP	0.094	0.38	0.5	1	This Study
DAP Pre-diffusion method (mm)		30	30	22	18	This Study
VAN PAP-AUC ratio		0.98	1.48	2.81	5.7	[24]
Phenotype		hVISA	hVISA	VISA	VISA	[24]

MIC: Minimal inhibitory concentration, VAN: vancomycin, OXA: oxacillin, RIF: rifampin, DAP: daptomycin, PAP-AUC: Population analysis profile and area under the curve.

**Table 2:** Accession numbers, genome coverage and assembly metrics of genomes included in this study. # Contigs is the total number of contigs in the assembly. # Contigs (≥x bp) is the total number of contigs of length ≥ x bp. Largest contig is the length of the longest contig in the assembly. Total length is the total number of bases in the assembly. Total length (≥x bp) is the total number of bases in contigs of length ≥ x bp. GC (%) is the total number of G and C nucleotides in the assembly, divided by the total length of the assembly. N50 is the length for which the collection of all contigs of that length or longer covers at least half (50%) the total base content of the assembly. N90 is used for the same purpose but the length is set at 90% of total base content instead of 50%. L50 is the number of contigs equal to or longer than the N50 length. L90 is used for the same purpose in reference to the N90 length. # N’s per 100 kbp is the number of ambiguous bases (Ns) per 100 kbp.

Assembly	D1	D2	D2P11	D23C9
Assembly accession	JAPZAH000000000	JAPZAI000000000	JAPZAJ000000000	JAPZAK000000000
Genome coverage	286.77	246.87	277.45	256.49
# Contigs (≥0 bp)	45	45	35	47
# Contigs (≥1000 bp)	38	38	31	40
Total length (≥0 bp)	2,795,159	2,795,155	2,785,355	2,787,646
Total length (≥1000 bp)	2,789,975	2,789,971	2,782,204	2,782,459
# Contigs	45	45	35	47
Largest contig	567,081	567,084	567,084	419,127
Total length	2,795,159	2,795,155	2,785,355	2,787,646
GC (%)	32.79	32.79	32.8	32.81
N50	334,617	334,619	337,832	148,220
N90	36,723	36,723	39,437	33,617
L50	4	4	4	5
L90	17	17	16	19
# N’s per 100 kbp	0	0	0	0

**Table 3:** Summary of genomic features shared by all strains. Genotypic features (MLST and SCC*mec* type), and the presence of full/complete genetic determinants (mobile genetic elements, antimicrobial resistance and restriction modification systems) detected in the genomes included in this study.

Feature	
MLST	ST100
SCC*mec* type	NT
AMR determinants	*aac(6′)-aph(2″)*, *blaZ*, *mecA*, *rpoB* H481N
Insertion sequences	IS*256*, IS*1181*, IS*431*, IS*Sau6*
Transposon	Tn*4001*
RM systems	S.Sau N315 I–M.Sau N315 I, S.Sau N315 II–M.Sau N315 II, SauUSI
Plasmidic *rep* genes	rep21.13_SAP101A (GQ900495.1), rep20.3_pTW20 (FN433597.1)

NT: Non-typeable. MLST: Multilocus sequence type. AMR: Antimicrobial resistance. RM systems: Restriction modification systems.

**Table 4:** Non-synonymous SNPs related to peptidoglycan metabolism or vancomycin reduced susceptibility shared among all strains analyzed in this study, after mapping the reads to the *S. aureus* N315 reference genome. All changes are expressed in reference to the *S. aureus* N315 genome.

Chromosome Position	Gene Name	Product	Predicted Amino Acid Change
46,301	*mecA*	Penicillin-binding protein 2 prime	Gly246Glu
297,502	*tarF_1*	CDP-glycerol:poly(glycerophosphate) glycerophosphotransferase	Asn236Ser
581,060	*rpoB*	DNA-directed RNA polymerase subunit beta	His481Asn
583,428	*rpoC*	DNA-directed RNA polymerase beta’ chain protein	Pro41Ala
1,595,731	*rpoD*	RNA polymerase sigma-70 factor RpoD	Val253Ile
687,696	*tagG*	Teichoic acid ABC superfamily ATP-binding cassette transporter	Val220Ala
732,508	*cydD*	ABC superfamily ATP-binding cassette transporter	Asn174Asp
902,157	*dltD*	D-alanine lipoteichoic acid and wall teichoic acid esterification secreted protein	Ile264Lys
993,796	*murE*	UDP-N-acetylmuramoyl-L-alanyl-D-glutamate--2	Ala436Ser
1,157,903	*ftsL*	Cell division and chromosome partitioning protein	Asp78Asn
1,203,919	*pknB_2(stk1)*	Non-specific serine/threonine protein kinase	Lys512Asn
1,487,245	*pbp2*	Glycosyl transferase family protein	Cys197Tyr
1,487,506	*pbp2*	Glycosyl transferase family protein	Thr284Ile
1,758,442	*ezrA*	Septation ring formation regulator	Val545Ala
2,009,774–2,009,775	*ami*	N-acetylmuramoyl-L-alanine amidase	Ala250Val
2,010,074	*ami*	N-acetylmuramoyl-L-alanine amidase	Asn150Lys
2,010,079	*ami*	N-acetylmuramoyl-L-alanine amidase	Ile149Val
2,138,921	*murF*	Putative UDP-N-acetylmuramoylalanyl-D-glutamyl-2	Ser126Asn
2,171,885	*rho*	Putative methicillin resistance expression factor	Ile48Leu
2,412,317	*tcaA*	Teicoplanin resistance-associated membrane protein TcaA protein	Leu218Pro

**Table 5:** Genetic changes differing between strains analyzed in this study after mapping the reads to the *S. aureus* D1 genome. All changes and positions are expressed in reference to the *S. aureus* N315 reference genome except otherwise stated. Genes without an annotated name are in reference to the CDS of N315 (or D1 genome if not present in the latter) by their locus tag.

Chromosome Position	Gene Name	Product	Type	Predicted Aminoacid Change			
				D1	D2	D23C9	D2P11
1,506,816	Intergenic (upstream *aroC*)	-	SNP	C63217A *	wt	wt	wt
1,003,592	Intergenic (upstream *comK*)	-	SNP	T19402C *	wt	wt	wt
170,209	*cap5D*	Capsular polysaccharide biosynthesis protein Cap5D	SNP	Thr215Ile	wt	wt	wt
1,201,763	*stp1*	Protein phosphatase 2C domain-containing protein	INDEL	Gly41_Lys43dup	wt	wt	wt
2,713,713	*nsaS/braS*	Integral membrane sensor signal transduction histidine kinase	INDEL	Gln63del	wt	wt	wt
1,832,949	*sagB*	Beta-N-acetylglucosaminidase	INDEL	Wt	His142fs	His142fs	His142fs
12,058(NODE_18) *^2^	SAD1_02353	Bacteriophage tail tape measure protein	SNP	Wt	Asp1737Gly	Asp1737Gly	Asp1737Gly
27,119(NODE 19) *^2^	SAD1_02404	GNAT family acetyl transferase	SNP	Wt	Tyr158Tyr	Tyr158Tyr	Tyr158Tyr
24,307(NODE 21) *^2^	*ccrB*	Cassette chromosome recombinase B	SNP	Wt	Asn10Ser	Asn10Ser	Asn10Ser
1,728,074	*phoR*	Alkaline phosphatase synthesis sensor protein PhoR	SNP	Wt	wt	Arg200 * STOP	wt
1,603,348	*era*	GTP-binding protein Era	INDEL	Wt	wt	wt	Asn81fs

* Intergenic change. wt: wild-type related to the N315 genome. *^2^ Position in D1 genome.

**Table 6:** Number of insertion sequences from Staphylococcus sp. per genome detected by ISseeker in the genomes included in this study. ISseeker identifies the termini of IS (>97% of identity) at contig edges and annotate flanking regions based on alignment of IS flanks with a reference genome.

IS	D1	D2	D23C9	D2P11
IS*256*	14	14	17	13
IS*1181*	7 + 1 *	7 + 1 *	7 + 1 *	7 + 1 *
IS*1182*	0	0	0	0
IS*1272*	1 *	1*	1 *	1 *
IS*431*	1	1	1	1
IS*Sau1*	0	0	0	0
IS*Sau2*	3 *	3 *	3 *	3 *
IS*Sau3*	10 *	10 *	10 *	10 *
IS*Sau4*	0	0	0	0
IS*Sau5*	1 *	1 *	1 *	1 *
IS*Sau6*	6	6	6	6
IS*Sau8*	2 *	2 *	2 *	2 *
IS*Sau9*	0	0	0	0
IS*Sep1*	1 *	1 *	1 *	1 *
IS*Sep2*	1 *	1 *	1 *	1 *
IS*Sep3*	0	0	0	0
IS*Sha1*	0	0	0	0

* Partial IS sequence inside a Contig, >97% Identity.

**Table 7:** IS*256* insertion sites detected with ISseeker software after annotating IS*256* flaking regions against the *S. aureus* N315 reference genome. Type was considered “intergenic” if IS*256* was found between two different genes, and “disrupting gene” if both annotated flanking regions were found in a single (same) gene.

Genomic Location	Type	D1	D2	D23C9	D2P11
*mecR1*	Disrupting gene	+	+	+	+
SA0142 (hypothetical protein)	Disrupting gene	+	+	+	+
SA0084 (hypothetical protein)—SA0085 (tRNA dihidrouridine sintase)	Intergenic	+	+	+	+
SA0516 (tRNA specific adenosine deaminase)—SA0517 (ABC superfamily ATP-binding cassette transporter)	Intergenic	+	+	+	+
*vraG* (ABC transporter permease)—SA0618 (*pitR*, putative phosphate uptake regulator) **	Intergenic	+	+	+ *	+ *
SA0621 (integral membrane protein) **—*rbf* (AraC type transcription regulator) **	Intergenic	+	+	-	-
*sarX* (staphylococcal accessory regulator protein X) **—SA0624 (putative transcriptional regulatory protein)	Intergenic	+	+	+ *	+ *
SA0625 (hypothetical protein)—SA0626 (hypothetical protein)	Intergenic	-	-	+	-
SA0742 (*clfA*, clumping factor A)	Disrupting gene	-	-	+	-
SA0954 (hypothetical protein)—SA0955 (hypothetical protein)	Intergenic	+	+	+	+
SA0185 (putative membrane protein YfhO)—*rbgA* (GTP-binding protein)	Intergenic	+	+	+	+
SA1176 (hypothetical protein)—*tkt* (transketolase)	Intergenic	+	+	+	+
SA1232 (*lysA*, diaminopimelate decarboxylase)—SA1233 (*msaC*, modulator of *sarA*) ***	Disrupting gene	+	+	+	+
SA1639 (hypothetical protein)	Disrupting gene	+	+	+	+
SA1648 (enterotoxin seO)—tRNAser	Intergenic	+	+	+	+
*agrB* (accesory gene regulator B)	Disrupting gene	-	-	+	-
SA2019 (hypothetical protein)—SA2010 (hypothetical protein)	Intergenic	+	+	+	+
SA2414 (hypothetical protein)—SA2415 (*braE*, ABC superfamily ATP-binding cassette transporter membrane protein)	Intergenic	-	-	+	-

+ Presence of IS*256* in the genomic location. - Absence of IS*256* in the genomic location. * IS*256* insertion site was detected but one of the genes in the flanking region is not present in the analyzed assembly, ** Genomic region encompassing *pitR* and *sarX* genes, absent in D23C9 and D2P11 (8 kb deletion), *** The annotated *msaC* gene was found deleted in all genomes analyzed in this study.

## Data Availability

Genomic reads and assemblies can be found in the National Center for Biotechnology Information (NCBI) genome database in the Sequence Read Archive (SRA) under the BioProject PRJNA911808 with the following biosample accession numbers: SAMN32204997 (D1), SAMN32205060 (D2), SAMN32205332 (D23C9), SAMN32205398 (D2P11). Whole Genome Shotgun project has been deposited at DDBJ/ENA/GenBank under the accessions JAPZAH000000000 (D1), JAPZAI000000000 (D2), JAPZAJ000000000 (D23C9), JAPZAK000000000 (D2P11).

## Supplementary Materials

The following supporting information can be downloaded at: https://www.mdpi.com/article/10.3390/antibiotics12020372/s1, Table S1: Oligonucleotides used to confirm chromosomal deletion and IS*256* insertions. Region amplified is referred to the annotation in *S. aureus* N315 reference genome. * amplicon was purified and sequenced; Table S2: Genomic features of the genomes analyzed in this study; Table S3: Core mutations present in the strains analyzed in this study found by Snippy core (not including INDELs) after mapping the reads to the *S. aureus* N315 reference genome.
